# *Momordica charantia* (Indian and Chinese Bitter Melon) Extracts Inducing Apoptosis in Human Lung Cancer Cell Line A549 via ROS-Mediated Mitochodria Injury

**DOI:** 10.1155/2019/2821597

**Published:** 2019-03-06

**Authors:** Siroshini Thiagarajan, Daryl J. Arapoc, Nurul Husna Shafie, Yong Yoke Keong, Hasnah Bahari, Zainah Adam, Thandar Ei

**Affiliations:** ^1^Department of Human Anatomy, Faculty of Medicine and Health Science, Universiti Putra Malaysia, 43400 Serdang, Selangor, Malaysia; ^2^Malaysian Nuclear Agency, 43000 Kajang, Selangor, Malaysia; ^3^Department of Nutrition, Faculty of Medicine and Health Science, Universiti Putra Malaysia, 43400 Serdang, Selangor, Malaysia

## Abstract

Lung cancer is the leading cause of cancer related deaths worldwide with about 40% occurring in developing countries. The two varieties of* Momordica charantia*, which are Chinese and Indian bitter melon, have been subjected to antiproliferative activity in human non-small cell lung cells A549. The A549 cells were treated with hot and cold aqueous extraction for both the bitter melon varieties, and the antiproliferative activity was evaluated by 3-(4,5-dimethylthiazol-2-yl)-2,5-diphenyltetrazolium bromide (MTT) assay. The apoptotic mechanism of action on A549 human lung cancer cells was evaluated first morphologically using Hoechst 33358, and cytoskeleton staining using Filamentous-actin (F-actin) cytoskeleton FICT and DAPI followed by caspase-3/7, reactive oxygen species (ROS), and p53 activity. Chinese hot aqueous extraction (CHA) exhibited potent antiproliferative activity against A549 human lung cancer cells. The morphological analysis of mitochondria destruction and the derangement of cytoskeleton showed apoptosis-inducing activity. CHA increased the caspase-3/7 activity by 1.6-fold and the ROS activity by 5-fold. Flow cytometric analysis revealed 34.5% of apoptotic cells significantly (p<0.05) compared to cisplatin-treated A549 human cancer cells. CHA is suggested to induce apoptosis due to their rich bioactive chemical constituents. These findings suggest that the antiproliferative effect of CHA was due to apoptosis via ROS-mediated mitochondria injury.

## 1. Introduction

Cancer is a form of silent disease characterized by uncontrolled and unscheduled cell proliferation. This heterogeneous disease causes normal cells to escape the cell regulation cycle forming a tumor[1]. Cancer is one of the major diseases in Malaysia. In 2017, cancer has been reported as the fourth most common cause of death in private hospitals in Malaysia (International Agency of Research for Cancer [IARC], 2018). The number of cancer cases showed a drastic change from from 37,400 in 2012 to 43,837 in 2018. [2].

Among them, non-small-cell lung cancer (NSCLC) is the leading cause of cancer death in the world. Unfortunately, current therapy is still inadequate, and the 5-year survival rate for lung cancer remains poor [3]. Plant and herbal-derived medicines have been used as traditional medicines since ancient times [4]. Late in 1950s the anticancer agents from plant-derived sources were discovered and brought to the evolution of vinca alkaloids, vincristine, and vinblastine [5].Bearing this in mind, the aim of this study is to explore the anticancer property of* Momordica charantia *on A549 human lung cancer cell line.

Most commonly used as a folk medicine,* M. charantia* has been subjected to having antiviral, antileukemia, anthelmintic, anticancer, and antidiabetic properties [6–8]. Commonly known as bitter melon or bitter gourd, this plant can be vastly cultivated throughout Asia, Africa, and also South America. It is not only easily cultivated, but a number of preliminary studies have also reported on the anticancer activity possessed by a crude extract of* M. charantia *[9]. Studies have also reported that the water-soluble extract of bitter gourd exerts anticancerous activity via inhibiting the cellular protein synthesis, RNA, and also DNA configurations.

The whole bitter gourd has been reported to have high total phenolic, total flavonoid, and saponin contents and relatively high antioxidant activity which have many health-promoting effects [6]. Flavonoids, the naturally occurring polyphenolic compound with complex pharmacological properties, are associated with lowering the cancer risk when consumed daily [7]. Furthermore, it has also been reported that bitter gourd contains gallic acid, p-coumaric acid, ferulic acid, catechin, caffeic acid, and tannic acid [8].

In this research, the whole fruit extract of* M. charantia *was used to determine the cytotoxic effect on human cancer cell lines and to investigate the underlying mechanism of apoptosis. Although there are many studies on* Momordica* genus, only a few studies reported on Malaysian species of* M. charantia.*

## 2. Materials and Methods

### 2.1. Plant Sample Preparation

Two types of* M. charantia* were used in this study, namely, the Chinese and the Indian bitter melon [10]. The samples were separated by washing over running tap water. Samples were then dried at 40°C in the oven for three days. Then, the dried samples were ground into a fine powder by sieving it over a fine mesh sieve. Samples were stored at -20°C prior to extraction.

### 2.2. Hot Water Extraction

The aqueous extract of samples was prepared by decoction. One hundred gram of dried powdered sample was soaked in 1 L of water at 70°C for 12 hrs [8]. The aqueous extract was filtered using a Whatman filter paper. The filtered extracts were then kept in -80°C prior to freeze-drying. The freeze-dried samples were stored at -20°C.

### 2.3. Cold Water Extraction

A 100 g of the dried leaves powder of samples was mixed with 1000 ml of distilled water and left at room temperature for 2 days [8]. The aqueous extract was filtered using Whatman filter paper No. 1 and stored in -80°C to be freeze-dried. The extracts were stored at -20°C until needed in the experiment.

### 2.4. Cell Culture

The human lung cancer cell line A549 was obtained from American Type Culture Collection (ATCC) and maintained in RPMI-1640 supplemented with 10% FBS and 1% penicillin-streptomycin at 37°C in a humidified atmosphere of 5% CO_2_. Cells were maintained by changing media every 2-3 days and subcultured until cells reached 70% confluency.

### 2.5. Measurement of Cell Viability by MTT Assay

The effect of* M. charantia *on the proliferation of A549 cells was measured using MTT assay. In brief, A549 cells were plated at 2.0 × 10^3^ cells per well in 96-well plate overnight [8, 9]. The cells were treated at a various concentrations of* M. charantia *crude extracts (1000 *μ*g/mL, 800 *μ*g/mL, 600 *μ*g/mL, 400 *μ*g/mL, 200 *μ*g/mL, and 100*μ*g/mL) for 24, 48 and 72 hrs. After incubation, 20 *μ*L MTT solution (5mg/mL) was added to each well and read for the absorbance at 570 nm (Microplate reader E16).

### 2.6. Morphological Analysis of Apoptosis by Hoechst 33358 Staining

A549 cells were seeded at 1 × 10^6^ cells per well in a six-well plate left to attach overnight. The cells were then treated with the IC_50_ of each crude extract of* M. charantia *for 24 hrs. After treatment, the cells were washed twice with 1 mL of cold PBS and fixed with 1 * *mL* *cold methanol and acetic acid at the 3:1 (v/v) for 30 mins in dark. The fixed cells were stained with 500 *μ*Lof Hoechst 33358 (1 mg/ml) for another 30 mins in dark [6]. Cells were observed using a fluorescence microscope (Microscope, Olympus DX51; Camera, Olympus DP72).

### 2.7. Fluorescence Actin Cytoskeletal Staining

Cells were seeded at 2.5 × 10^3^ cells per well in a 6-well plate on a glass culture slide. The cells were then treated with* M. charantia* extracts and also cisplatin as a positive control. After treatment for 24 hrs, the cells were fixed with 2 mL of 3.7 % paraformaldehyde for 10 mins. The cells were washed with 2 mL of PBS twice and then permeabilized with 2 mL of 0.1 % Triton-X for 5 mins. The cells were then washed twice with PBS and stained with Fluorescence Phalloidin (SKU F432) for 20 mins at room temperature, followed by 0.5*μ*g/mL DAPI staining for 5 mins [8]. Lastly, cells were washed with PBS for another 5 mins. Cells were observed using a fluorescence microscope (Microscope, Olympus DX51; Camera, Olympus DP72).

### 2.8. Detection of Intracellular Reactive Oxygen Species (ROS) Levels

Lung cancer cells A549 were seeded at 2.5 × 10^3^ cells per well in a black clear bottom 96-well plate and then treated with* M. charantia* extracts and cisplatin as a positive control for 24 hrs. Cells were then incubated with 5*μ*M DCFH-DA at 37°C for 30 mins [7]. The plate was read using an excitation 480nm and emission 520nm (EnSpire Multimode Plate Reader, PerkinElmer).

### 2.9. Measurement of P53 Suppressor Activities

Cells were seeded at 2.5 x 10^3^ cells per well in a 96-well plate and treated at the IC_50_ crude water-soluble extract of* M. charantia *for 24 hrs. Conditioned media and the lysate of the treated cells were obtained, and p53 activities were measured using the Human p53 ELISA Kit (NovaTeinBio, Boston) according to the manufacturer's instructions.

### 2.10. Measurement of Caspase-3/7 Activities in Treated and Untreated A549 Cells

Caspase 3/7 activities were measured using the Apo-ONE® Homogeneous assay kit (Promega, USA) according to the manufacturer's instructions. Lung cancer cells were seeded at 2.5 x 10^3^ and incubated at the IC_50_ of a different crude water-soluble extract of* M. charantia *and also a nontreated lung cancer cells. The caspase-3/7 activity was done according to the manufacturer's protocol. A hundred microliters of Apo-ONE® caspase-3/7 reagent was added to a 96-well plate which contains blank, control, and also treated cells. Contents were gently mixed using a plate shaker at 300rpm until reading time to 4 hours. The fluorescence of each well was measured with an optimal excitation wavelength 499 nm and an emission wavelength of 521 nm. The measurements were done empirically for 10 mins interval within 4 hrs (EnSpire Multimode Plate Reader, PerkinElmer).

### 2.11. Flow Cytometric Analysis of Apoptosis

A549 cells were seeded at 5.0 × 10^3^ cells per well in a 96-well plate. The cells were treated the day after for 24 hrs. Prior to imaging, 100 *μ*L of Hoechst 33342 was added to the final concentration of 10 *μ*g/ml and further incubated for 30 mins [7]. Data were then exported functionality within CellReporter software, where the cell counts for live and dead cell populations were tabulated in a histogram plot.

### 2.12. Statistical Analysis

All data were presented as means ± SEM of three independent experiments carried out in triplicate. Statistical analysis was performed by using one-way analysis of variance (ANOVA) Tukey's post hoc test, IBM SPSS statistics version 22.0. A p-value less than 0.05 (p<0.05) was regarded as statistically significant.

## 3. Results

The cell lines were incubated with the extracts for 24, 48, and 72 hrs. The results show that A549 with the crude water-soluble extract evoked significant (p <0.05) decrease in cell viability compared to the untreated cells. From the time-course experiment, it was established that the IC_50_ decreased to its maximum level at 24 hrs of treatment. Therefore, the treatment time of 24 hrs was subjected to all the subsequent experiments of this study. It was observed that both the crude water-soluble extracts had little or no effect on the death of normal human lung cell line, MRC5.

Table 1 shows the results of A549 cell treated with crude extracts for 24 hrs. The cell proliferation of each tested group was determined by MTT assay. The IC_50_ of cisplatin used as a positive control for A549 cell under the same experimental procedure was 17.3 ± 0.01*μ*g/mL. Among all the extracts, ICA exhibited the lowest IC_50_ of 26.7 ± 0.05 *μ*g/mL followed by CCA (28.1 ± 0.19 *μ*g/ml), CHA (32.5 ± 0.18 *μ*g/ml), and IHA (36.9 ± 0.08 *μ*g/ml). All data were shown as mean ± SEM, different experiments in triplicate.

Table 1 shows the results of A549 cell treated with crude extracts for 24 hrs, and the cell proliferation of each tested group was determine by MTT assay. Results were expressed as mean ± SEM of three independent experiments performed in triplicate.

**Figure 1:**
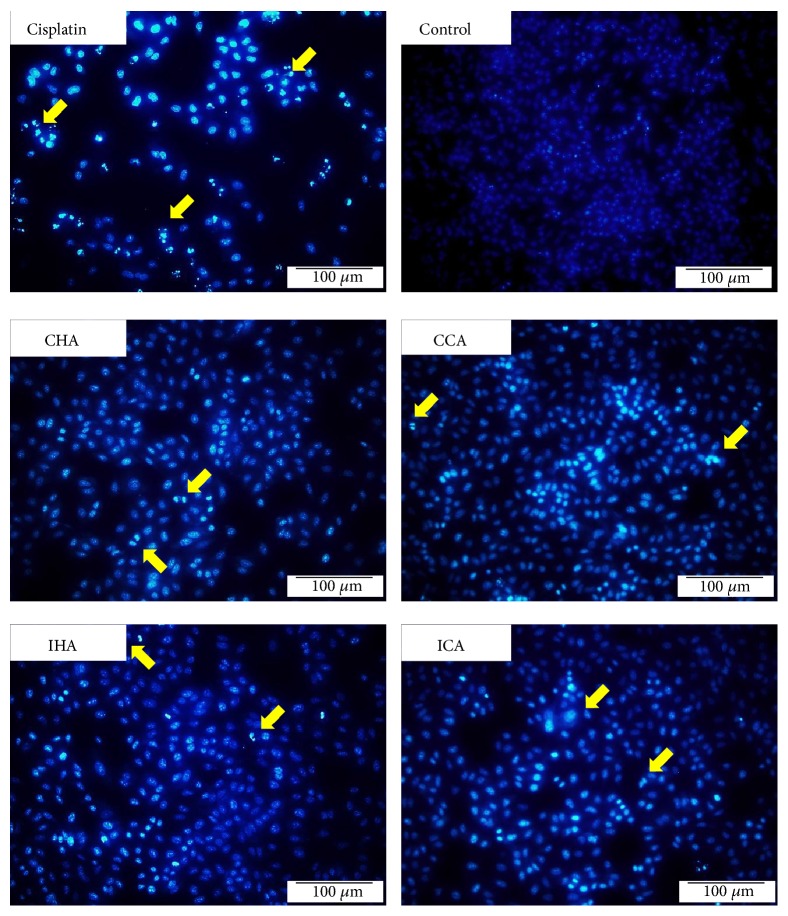
Cell apoptosis observed using Hoechst 33358 staining. A549 cells were treated with* M. charantia* extracts for 24 hrs. Treated A549 cells exhibited morphological changes in the nuclei with stronger blue fluorescence (typical of apoptosis) than non-apoptotic cell. Photographs were taken under a fluorescence microscope (200×, original magnification). The yellow arrows represent apoptotic cells.

To confirm the apoptosis-inducing effect of the extracts on the cancer cells, the cells were stained with Hoechst 33358. Nuclear morphological changes under fluorescence microscope were assessed and the apoptotic cells are indicated by arrows seen in Figure 1. The apoptotic cells revealed highly fluorescent condensed chromatin. Treated cells showed condensed, fragmented, and small nuclei which have typical apoptotic morphology in comparison to the blue symmetrical normal nuclei. Along with the condensed chromatin, the flow cytometry result has revealed that the percentage of apoptotic cells treated with cisplatin caused 41.26 ± 2.77%; control: 2.63 ± 0.47%; CHA: 34.50 ± 7.71%; CCA:32.04 ± 5.87%; IHA: 32.50 ± 4.20%; and ICA: 37.85 ± 2.61% cell death.

**Figure 2:**
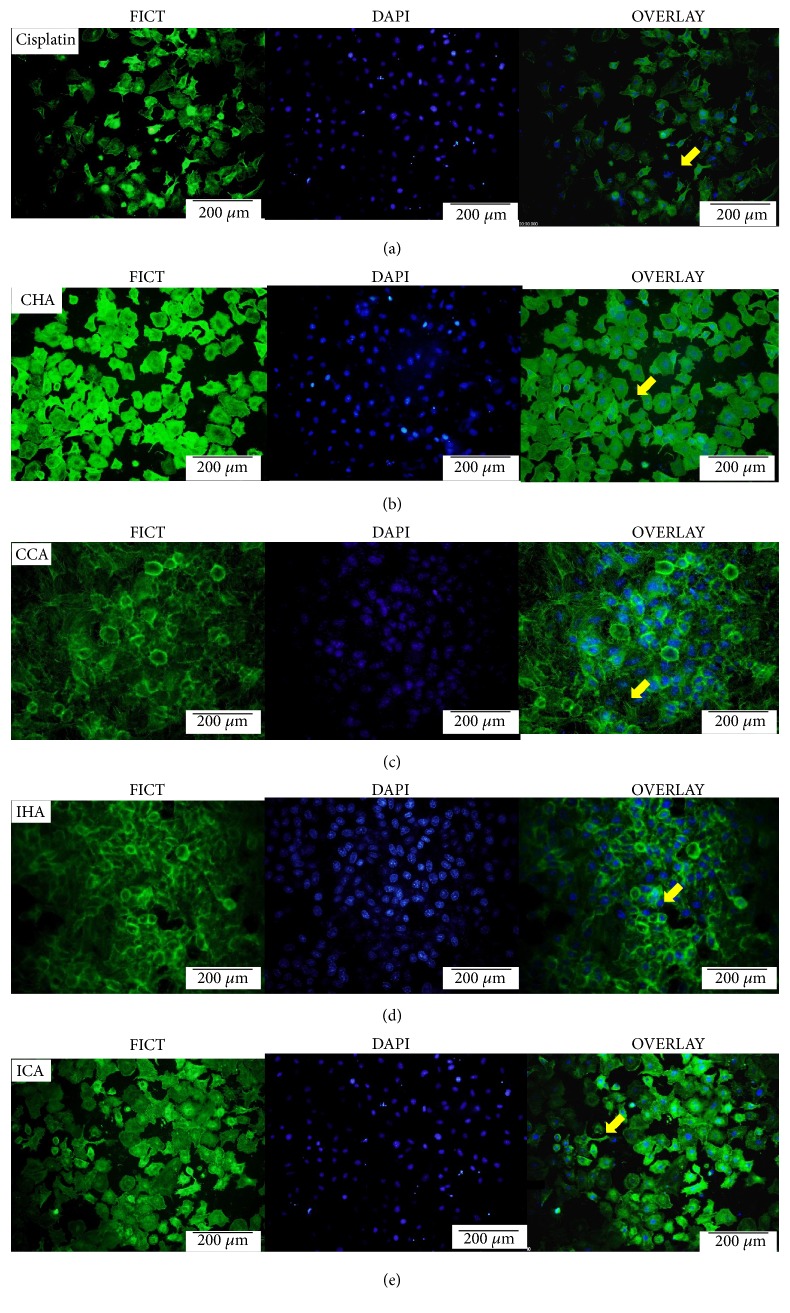
The effects of* M. charantia* on the expression of F-actin and nuclear membrane under 20× magnification. Cisplatin caused derangement of F-actin fibres and nuclear condensation indicated by the yellow arrow. All cells showed a derangement in F-actin and mitochondria disruption which was stained green and blue color, respectively. (Scale bar= 200*μ*m.)

The change in epithelial cell shape leads to the detachment of cells which causes apoptosis. Actin is an important component in different processes including cell growth and also cell death. The nuclear membrane degradation showed condensed and fragmented nuclei under the microscope. All cells treated with* M. charantia* crude extract showed destruction and the alteration of actin and nuclei of the cells. However, massive destruction of actin filaments and the nuclei can be seen in Figure 2 in the cell treated with cisplatin. The degree of destruction reduces when A549 cells were treated with CHA and ICA compared with cisplatin.

**Figure 3:**
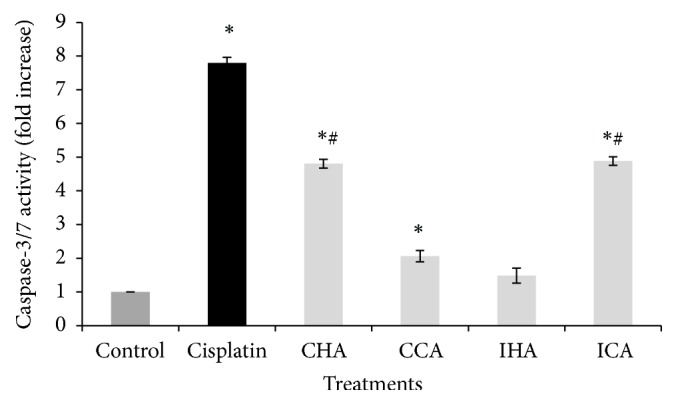
The effect of crude extracts on caspase-3/7 activity (fold increase) from treated A549 cells. The level of caspase-3/7 release was determined using the EnSpire Multimode Plate Reader, PerkinElmer, at the emission and an excitation wavelength of 499/521 nm. Data are presented as mean ± SEM, ^*∗*^p<0.05, compared with control, and ^#^p<0.05 compared with cisplatin.

To confirm the apoptosis event, caspase-3/7 activity was accessed on A549 cells. The results show that caspase-3/7 activity shown in Figure 3 in the untreated cell lines decreased compared to cisplatin. It can be seen that the positive control (cisplatin) showed the highest (8-fold increase, p<0.05) caspase activity when compared to the untreated cells. The cells treated with the crude extract ICA, CCA, and CHA showed a significant (p<0.05) pronounced increase in caspase-3/7 activity compared to untreated samples. The CHA and ICA showed an increase in caspase-3/7 activity at 61.64% and 62.68%, respectively, when compared to the untreated cells normalized to 100%.

**Figure 4:**
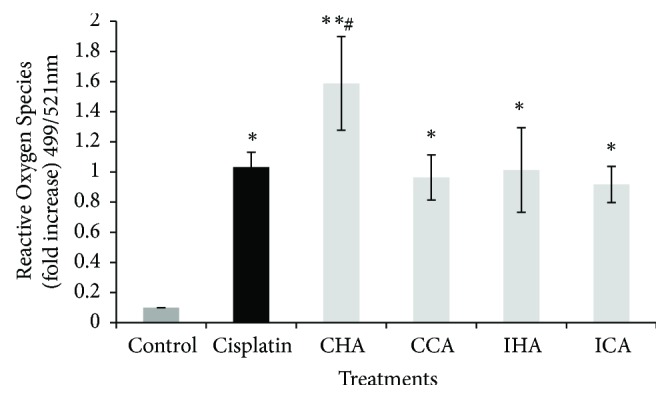
Quantitative measurement (fold increase) of the expression of Reactive Oxygen Species (ROS) level on treated A549 cells. Results are presented as mean ± SEM (n=3); ^*∗*^p<0.05 and ^*∗∗*^p<0.01 compared with control; ^#^p<0.05 compared with cisplatin.

ROS reduces cell viability, triggers the ROS generation, and thereby results in cell death. It can be observed that cisplatin triggers 1.2-fold increase when compared to the untreated cells (Figure 4). However, CHA showed the highest (1.58-fold) and significant ROS generation (p<0.01) followed by IHA (1.01-fold), CCA (0.96-fold), and ICA (0.91-fold) increase compared to the untreated cells.

**Figure 5:**
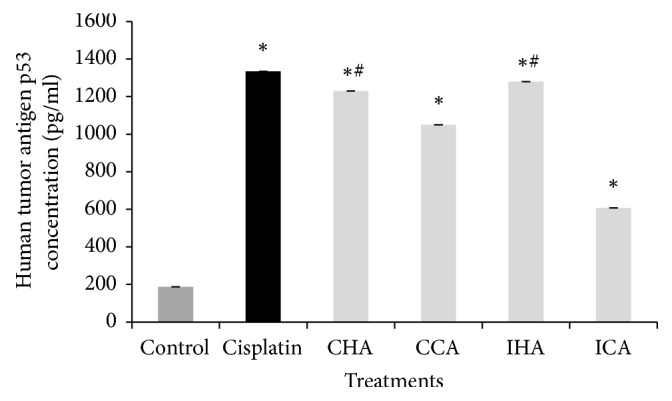
Human cellular tumor antigen p53 concentration (pg/ml) in A549 cell lysate conducted using the Human p53 ELISA Kit (NovaTeinBio, Boston). Data are expressed as concentration (pg/ml) and the value represents the mean ± SEM (n=3) of three independent experiments. ^*∗*^p<0.05 compared with control, and ^#^p<0.05 compared with cisplatin.

In return of mitochondria disruption and increase in ROS production, crude extratcs were analysed for the p53 activity. As shown in Figure 5 the p53 activity expressed in the cell lysate was the highest in cisplatin. The concentration of p53 in cisplatin-treated cells was 1400 pg/ml significant (p<0.05) compared with the control. All the crude water-soluble extracts showed a significant increase when compared with control. When compared to cisplatin, only CHA and IHA treated cell showed significance (p<0.05). The concentration of p53 in IHA treated cells was the highest (1300 pg/ml) followed by CHA (1200 pg/ml). When comparing between the same variations of bitter melon, the hot extraction showed higher expression of p53 compared to the cold extraction of both Chinese and Indian bitter melon.

## 4. Discussion

Numerous plants have been identified and used as a treatment for different diseases throughout the world, mostly in underdeveloped countries. Researchers have been focusing on the scientific evaluation of traditional drugs obtained from equatorial plant, namely,* M. charantia*, which has been popularly used among cancer and diabetic researchers. Commonly known by the locals as either bitter gourd or bitter melon, it is being described as the food of medicine [8]. This plant can be harvested all around Africa, Asia, and South America. Being closely related to cucumber and also squash, it grows in a slender climbing annual vine with long-stalked leaves along with yellow, solitary male and female flowers borne in the leaf axils [11]. Cancer therapies are usually based on surgery, chemotherapy, and radiotherapy. However, some of the patients are not fully cured [12, 13]. Therefore, natural products are a better alternative for the treatment of cancer without any serious side effects.

The viability assay was used to determine the suitable dose (IC_50_) and the possible cytotoxic effect of the crudes extracts on A549 cells. The cytotoxicity in each crude extracts showed a significant decrease in cell viability when compared to untreated cells at 100% viability. A known chemotherapy drug, cisplatin, was used as a positive control in this study and showed an IC_50_ of 17.3 ± 0.01* μ*g/ml which had the strongest inhibition to A549. A study by Manoharan et al., 2014, resulted in 800 *μ*g/ml of crude extract on A549 cells at the time course of 24 hrs, while our study shows a lower IC_50_ which indicates that the crude extracts had a better killing effect in the same period of incubation.

To further examine the mechanism of A549 cancer cell death induced, the Hoechst 33358 reagent was used to stain the DNA of the cells. Apoptotic cells were easily distinguished by the condensation and fragmentation of DNA, while nonapoptotic cells showed chromatin with an even, flat disc-like morphology. In contrast, apoptotic cells exhibited an uneven morphology consisting of a cluster of comparatively bright fragments of condensed DNA [14–16]. This was similarly seen in the study by Boo et al., 2011, and Jiang, Wu, Bai, Zhang & He, 2014, deliberating that the features observed are the traits during the early stage of apoptosis. Extracts cause morphological changes due to the active process in the endonuclease causing internucleosomal DNA to cleave and produce 180-200 base pair DNA fragments. In addition, the effects of* M. charantia* expression of F-actin and the nuclear membrane were observed. The derangement of F-actin was obvious in all treated cells. The surface of the epithelial cell will eventually turn apoptotic when being dissembled from their respective basement membrane [17]. The F-actin or cytoskeleton transformation will promote cell death via apoptosis-like pathway [18–21]. Moreover, the dysfunction of mitochondria as a result of mitochondria actin interactions also plays a role in the cellular morphology and adhesion of the cell [22].

Recently, ROS signaling became the focus of research on lung cancer as well as other cancer therapies. In recent lung cancer therapies, targeting ROS signaling was thought to be a striking method [23]. Few studies have shown that some herbal extracts and their components can eliminate active oxygen in lung cancer cells. The increase in ROS may be due to the oxidative phosphorylation uncoupling, hyperbaric O_2_ treatment, ischemia, and alterations of the mitochondrial lipids. The ROS signaling was prominent in all treated cells. The increase in ROS signaling is important to make sure that the cells were disrupted causing apoptosis. This is supported by Redza-Dutordoir & Averill-Bates, 2016, where a lower dose of ROS will induce a response in cell survival but a higher dose would stimulate apoptosis. This mitochondrial dysfunction may result in the rise of ROS activity which will successively cause cancer cell apoptosis through the mitochondrial stress pathway.

In return of mitochondria disruption and increased ROS production, crude extracts contribute to the p53-dependent apoptosis implying a mitochondrion-dependent pathway and also an important role in ROS-mediated apoptosis. The upregulation of p53 may be due to the disruption of outer membrane mitochondria under the influence of ROS initiating apoptosis. The tumor suppressor gene p53 plays a key role in the biological return to genotoxicity. The p53 can lead to either the onset of apoptosis or alternately initiate the DNA repair completing a cell cycle. It was suggested that bitter melon extracts can cause apoptosis by activation of p53 [9, 24, 25]. The p53 signaling activation can promote intrinsic pathway via the outer membrane mitochondria permeabilization. The expression of p53 activity in the cell lysate was generally high in three extracts, IHA, CHA, and CCA, compared to cisplatin, making them potential apoptosis agents via p53. The result obtained is supported by Amaral et al., 2010, where the tumor suppressor gene of p53 when activated will lead to the onset of apoptosis.

When compared to the caspase-3/7 activity, the CHA showed the highest expression at 1.6-fold followed by IHA (1-fold), CCA (0.9-fold), and lastly ICA (0.8-fold). The contradiction between IHA extract on ROS and caspase 3/7 activity has led to the conclusion that the cells could go through extrinsic apoptotic pathway as the p53 signaling results showed a low signal (600 pg/ml). This result is supported by the study of Li et al., 2012, where the* M. charantia *extract increased the caspase activity and caused cleavage of the cellular protein, causing apoptosis.

All extracts promoted apoptosis; however, the highest was seen in CHA treated A549 cells. ROS-mediated mitochondrial injury pathway suggested by the treatment of extract CHA is more significant with 34.5% of apoptotic cells. With the previous parameters, it can be postulated that the cells treated with* M. charantia *possess anticancer properties by inducing apoptosis of the A549 cells. These results are in agreement with previous studies where curcumin, MCME,* Morinda Citrifolia*, and other* M. charantia *extracts cause apoptosis with the changes in cell morphology and the increase in ROS activity and caspase-3/7 activity, followed by the p53 activity [9, 26, 27].

Due to the persistent anticancer activity subjected by the crude extracts on A549 cells, liquid chromatography-mass spectrometry (LC-MS) was carried out. The LC-MS resulted in several active compounds which can be isolated from the crude extract which can be the possible active compound for apoptosis in A549. The compound which is predominant at the highest yield was Camphor found in CCA while Norharman is found in ICA, IHA, and CHA crude extracts. Camphor is a monoterpene which is pharmaceutically used as an antiseptic, topical analgesic, antipyretic, anti-inflammatory, contraceptive, cough suppressant, and anticancer [28]. Camphor has two mechanisms of action which are chemoprevention and chemotherapy. The chemoprevention action occurs during the initiation phase of carcinogenesis to prevent the interaction of chemical carcinogens with DNA, by induction of phase I and phase II enzymes to detoxify the carcinogen [29]. As for chemotherapy, camphor works during the promotion phase, in which inhibition of tumor cell proliferation, acceleration of the rate of tumor cell death, and/or induction of tumor cell differentiation may occur [30].

Norharman which is known as a tricyclic indole *β*-carboline alkaloid has a simple chemical structure. Various pharmacological effects have been reported such as having anticancer properties [30, 31], bioactive interactions with monoamine oxidase type [32], inhibition of nitric oxide synthase and indoleamine 2,3-dioxygenase [32], and also improvement of the insulin secretion from human islets of Langerhans [32]. However, in the approach of anticancer studies, norharman has been tested with human tumor cells resulting in cytotoxic activity [33]. Norharman is said to interact with DNA, unwind the double helix by intercalation, and break DNA strands [34, 35].

## 5. Conclusion

**Figure 6:**
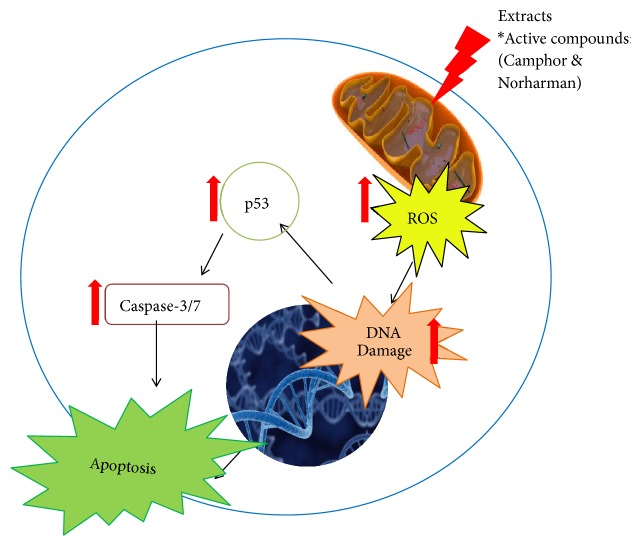
Mechanistic dissection.

Cisplatin had a better potency compared to the extracts in inducing apoptosis. As cisplatin is the standard drug for non-small cell lung cancer, it induces apoptosis by increasing the ROS via the intrinsic pathway of apoptosis. The antiproliferative effects of CHA in A549 cells were associated with mitochondria disruption, and induction of apoptosis by increasing ROS and caspase-3/7 activity as illustrated in Figure 6. These results suggest that CHA is an effective natural product for the treatment of lung cancer through the activation of ROS-mediated mitochondria injury.

## Figures and Tables

**Table 1:** Cytotoxic effect [IC_50_ (*μ*g/mL)] of Momordica charantia extracts and standard drugs against A549 cell and MRC5 after 24 hrs incubation.

			Cancer Cell Line	Normal Cell Line
	*M. charantia*	Solvents	A549	MRC5
Plant extracts	Chinese (C)	Hot Aqueous (HA)	32.5 ± 0.18	>500
		Cold Aqueous (CA)	28.1 ± 0.19	>500
	Indian (I)	Hot Aqueous (HA)	36.9 ± 0.08	>500
		Cold Aqueous (CA)	26.7 ± 0.05	>500
Standard drug	Cisplatin		17.3 ± 0.01	

## Data Availability

The data used to support the findings of this study are available from the corresponding author upon request.
